# Phage-Based Vaccine Platforms for Typhoid Fever: A Transformative Approach to Global Health and Antimicrobial Resistance

**DOI:** 10.3390/vaccines14100869

**Published:** 2026-10-01

**Authors:** Tambe David Tipah, Nnaemeka Emmanuel Nnadi, Roderick A. Slavcev

**Affiliations:** 1 Department of Microbiology, Faculty of Natural and Applied Sciences, Plateau State University, Bokkos 932112, Plateau State, Nigeria; tambetipah1@gmail.com; 2 Center for Phage Biology and Therapeutics (CENPBAT) LTD/GTE, Jos 930105, Plateau State, Nigeria; 3 School of Pharmacy, University of Waterloo, 10A Victoria St S, Kitchener, ON N2G 1C5, Canada; 4 Theraphage, 151 Charles St W Suite # 199, Kitchener, ON N2G 1H6, Canada

**Keywords:** typhoid fever, *Salmonella* Typhi, bacteriophage vaccines, phage display, DNA vaccines, typhoid conjugate vaccine, antimicrobial resistance, vaccine development, vaccine platforms, global health

## Abstract

Typhoid fever, caused by *Salmonella enterica* serovars Typhi and Paratyphi, remains a major public health threat across Africa and Asia and causes substantial annual morbidity. Transmission persists in low- and middle-income countries (LMICs), where access to safe water and sanitation is limited. While improvements in water, sanitation, and hygiene (WASH) are essential, slow implementation makes vaccination the most practical preventive strategy. Current licensed vaccines have limitations, including suboptimal immune coverage in infants, limited duration of protection, and cold-chain requirements. This review examines bacteriophage-based vaccine platforms as emerging preclinical strategies for typhoid prevention. Phage technologies, including phage display, phage DNA, and hybrid models, offer potential advantages such as multivalent antigen display, projected thermostability, and cost-efficient production using bacterial manufacturing systems. Based on animal immunogenicity and proof-of-concept studies, these systems could theoretically induce durable immunity and broaden age-group protection, although their clinical feasibility and scalability remain unproven. We evaluate key technical hurdles, including endotoxin contamination, host-strain engineering, and payload-expression constraints. We also discuss how these potentially low-cost platforms may contribute to global antimicrobial resistance (AMR) mitigation efforts. Although phage-based vaccines remain in the preclinical stage, they represent promising research platforms for advancing vaccine equity and infectious disease resilience, provided regulatory and technical challenges are overcome. However, significant translational gaps must still be bridged before clinical application can be realized.

## 1. Introduction

Typhoid fever, a systemic infection caused by *Salmonella enterica* serovars Typhi (*S.* Typhi) and Paratyphi (*S.* Paratyphi), remains a major global health burden, causing an estimated 12–21 million infections and up to 161,000 deaths annually, with the greatest burden in South Asia, Sub-Saharan Africa, and parts of the Middle East and Latin America [1,2,3]. School-aged children and adolescents bear a disproportionate burden due to frequent exposure to contaminated food and water, while younger children (<5 years) face higher risks of severe complications, such as intestinal perforation [4,5]. Transmission is further amplified by weak health systems in LMICs, where reference diagnostic tools like blood culture are rarely available, leading to frequent misdiagnosis, empirical antibiotic overuse, and underreported disease incidence [6,7]. Furthermore, the direct and indirect financial costs of prolonged illness place substantial economic strain on vulnerable households and national healthcare budgets [8].

The public health threat of typhoid is severely compounded by the rapid dissemination of multidrug-resistant (MDR) and extensively drug-resistant (XDR) *S.* Typhi strains. Resistance to traditional first-line agents, fluoroquinolones, and third-generation cephalosporins has forced reliance on costlier second-line antimicrobials like azithromycin and carbapenems [9,10]. With few new antibiotic classes in clinical development [11], vaccination is the most critical strategy to control disease burden and mitigate antimicrobial resistance (AMR). Although current typhoid conjugate vaccines (TCVs) offer superior immunogenicity over older Vi polysaccharide and Ty21a formulations, persistent challenges surrounding cold-chain dependence, procurement costs, manufacturing scalability, and equitable distribution highlight the need for next-generation vaccine platforms tailored for resource-limited settings.

Bacteriophage-based platforms encompassing phage display, phage DNA, and hybrid vector models represent an innovative approach to address these deployment barriers. Propagated via standard bacterial fermentation, phages offer a scalable, cell-free manufacturing paradigm [12,13,14,15,16]. Additionally, their structural organization permits high-density, multivalent antigen presentation and engineered surface modifications, while certain capsid architectures exhibit inherent physical stability [17,18,19]. However, while these features make phages an experimentally attractive platform, their proposed advantages regarding cold-chain independence, cost savings, and broad antigenic coverage require systematic validation under realistic deployment conditions.

Importantly, an expanding body of preclinical, target-animal, and field-trial evidence across diverse human and veterinary pathogens underscores the genuine translational potential of phage display technology. On T4 bacteriophage platforms, a dual-antigen nanoparticle presenting protective antigens against *Bacillus anthracis* and *Yersinia pestis* conferred 100% protection against lethal inhalational anthrax and pneumonic plague in animal models without external adjuvants [20]. Against viral and mucosal pathogens, CRISPR-engineered T4 nanoparticles displaying conserved influenza M2e repeats elicited long-lasting lung-resident memory CD4+ T cells and mucosal IgA, providing broad protection against divergent influenza strains [21]. Similarly, adjuvant-free dual T4 vaccines co-displaying SARS-CoV-2 spike trimers and influenza HA/M2e epitopes achieved complete protection against lethal dual viral challenges [22], while T4 constructs targeting Porcine Epidemic Diarrhea Virus (PEDV) generated high neutralizing antibody titers [23]. In bacterial systems, T4 nanoparticles displaying outer membrane protein C (OmpC) epitopes provided cross-serotype protection against extraintestinal pathogenic *Escherichia coli* (ExPEC) in mice, significantly reducing pulmonary and neurological bacterial loads [24].

Phage platforms have also demonstrated scalability and efficacy in large, natural target species under field conditions. Filamentous M13 phages displaying protective peptide pools provided robust cellular immunity against *Taenia solium* cysticercosis in swine [25], and a randomized field trial of over 1000 rural pigs in Mexico confirmed that a heat-inactivated M13 tripeptide vaccine (S3Pvac) significantly reduced manufacturing costs while decreasing total parasite burden by 87% [26]. Furthermore, recombinant T4 nanoparticles presenting multi-epitopes from elongation factor IF-2 elicited cross-serotype protection against *Streptococcus suis* in swine via needle-free administration routes [27].

At present, no bacteriophage-based vaccine has entered clinical trials for typhoid prevention, and evidence supporting phage platforms is derived from surrogate enteric models, platform-level studies, and non-typhoidal pathogens. Therefore, these findings serve as vital proof-of-concept for the underlying technology rather than demonstrated clinical efficacy against *S.* Typhi. This review examines bacteriophage-based vaccine systems as an emerging preclinical platform for typhoid prevention. It evaluates their principal design strategies, mechanisms of immune activation, and available preclinical evidence, while critically addressing the technical, manufacturing, biosafety, and regulatory barriers that must be overcome for clinical translation.

## 2. Current Vaccine Strategies

Vaccination remains the most practical and effective approach for preventing typhoid fever, particularly in LMICs where improvements in WASH are limited [28,29]. Multiple vaccines have been developed over recent decades, but their efficacy, duration of protection, and scalability vary [30,31,32,33,34,35]. Understanding the strengths and weaknesses of existing vaccines is essential to identify where novel platforms, such as bacteriophage-based technologies, could play a transformative role.

### 2.1. Conventional Vaccines

The earliest licensed typhoid vaccines include the live-attenuated oral Ty21a vaccine and the injectable Vi capsular polysaccharide (ViPS) vaccine. Although both vaccines have demonstrated protective effects against typhoid fever, they differ substantially in their biological mechanisms, immune responses, dosing requirements, and suitability for different age groups.

Ty21a is a live-attenuated oral vaccine derived from *S.* Typhi Ty2 and developed through chemical mutagenesis, including disruption of the galE gene involved in galactose metabolism [36,37]. The resulting attenuated organism retains sufficient biological activity to interact with the intestinal immune system while having reduced capacity to cause systemic disease. Following oral administration, Ty21a interacts with the intestinal mucosa and induces both systemic and mucosal immune responses [38]. Experimental studies in vaccinated adults have demonstrated Ty21a-specific CD4+ and CD8+ T-cell responses within the intestinal mucosa, particularly in the duodenum, approximately 18 days after vaccination [39]. These responses include production of multiple effector cytokines, including interferon-γ (IFN-γ), tumor necrosis factor-α (TNF-α), and interleukin-2 (IL-2), indicating a polyfunctional cellular immune response. The detection of antigen-specific T cells at the intestinal site is particularly relevant because *S.* Typhi is acquired through the gastrointestinal tract and subsequently crosses the intestinal barrier to establish systemic infection. Ty21a vaccination has also been associated with serum antibody responses against *S.* Typhi and *S.* Paratyphi antigens, including lipopolysaccharide (LPS), and mucosal cellular responses have been reported to correlate with systemic anti-*S.* Typhi IgG and IgA responses [40].

The immunological activity of Ty21a therefore extends beyond the induction of circulating antibodies and includes intestinal mucosal immunity and cell-mediated responses, which may contribute to protection against invasion and systemic dissemination. Studies have demonstrated the presence of vaccine-responsive CD4+ and CD8+ T cells expressing intestinal-homing markers such as integrin β7/α4β7, supporting the capacity of oral Ty21a vaccination to generate lymphocytes capable of trafficking to intestinal tissues [40]. However, the precise correlates and mechanisms responsible for protection remain incompletely defined. This is important because immunological responses observed following vaccination should not necessarily be interpreted as direct surrogates for clinical protection.

Clinical studies nevertheless demonstrate moderate protective efficacy of Ty21a. Three oral doses administered on days 0, 2, and 4 have been used in clinical studies, with protection demonstrated in both controlled human challenge studies and field trials [40]. Reported estimates of efficacy have varied according to population, study design, geographical setting, and follow-up period. A systematic assessment of available studies has reported approximately 35% efficacy in a UK human challenge study [41], 62% in a Chilean field trial [35], and 42.2% in an Indonesian field trial [42], illustrating the variability of protection observed across different epidemiological contexts. Other analyses have estimated approximately 48% cumulative protection over three years following a three-dose regimen [43]. The variability in estimates suggests that Ty21a provides meaningful but incomplete protection and that its effectiveness may depend on vaccination schedule, population characteristics, background exposure, and local epidemiology.

Despite these immunological and clinical benefits, Ty21a has several important limitations. Its live-attenuated nature, together with the need for oral administration and multiple doses, complicates implementation in some populations and vaccination programs. As with other live-attenuated formulations, Ty21a is generally contraindicated in significantly immunocompromised individuals due to the theoretical risk of uncontrolled bacterial replication and systemic infection [44,45]. Its performance is also less satisfactory in younger children, limiting its usefulness in populations in which early childhood represents an important period of typhoid exposure. The requirement for multiple doses may further reduce adherence and increase program complexity compared with single-dose or fewer-dose injectable vaccines. Thus, although Ty21a provides both mucosal and systemic immune responses and has demonstrated moderate clinical efficacy, its age restrictions, dosing requirements, and live-vaccine characteristics constrain its use as a universally applicable typhoid vaccine.

Vi polysaccharide (ViPS) vaccine represents a different approach based on administration of purified Vi capsular polysaccharide, typically as a 25-µg injectable dose [46]. Unlike Ty21a, ViPS does not contain a live organism and therefore avoids the safety limitations associated with live-attenuated vaccination. Its principal immunological mechanism is T-cell-independent activation of Vi-specific B cells [47,48]. The repetitive polysaccharide structures can cross-link B-cell receptors and stimulate antibody production without requiring conventional T-helper-cell assistance. Consequently, the major measurable immune response following ViPS vaccination is the production of serum anti-Vi antibodies, predominantly IgG [49,50]. However, because pure polysaccharide antigens trigger T-cell-independent immune responses, they induce weak immunological memory and a limited capacity for booster responses compared with protein-conjugated polysaccharide vaccines [51].

Clinical immunogenicity studies demonstrate that ViPS can produce a rapid and substantial antibody response following a single dose. In a study of individuals aged 2–16 years, vaccination produced a geometric mean anti-Vi antibody concentration of 1428.3 ELU/mL at six weeks, compared with 85.9 ELU/mL in controls (*p* < 0.001) [47]. Overall, 95.1% of vaccine recipients achieved antibody concentrations ≥ 150 ELU/mL, while 86.7% experienced at least a four-fold increase in antibody concentration from baseline. However, the response declined considerably over time. Two years after vaccination, the overall geometric mean concentration had decreased to 262.6 ELU/mL, with only 56.3% of participants retaining concentrations ≥ 150 ELU/mL and 39.1% maintaining at least a four-fold increase over baseline [47]. These findings demonstrate the relatively rapid waning of the antibody response following unconjugated Vi vaccination.

Age-specific differences further illustrate the limitations of the ViPS platform. Among children aged 5–16 years, the six-week geometric mean antibody concentration reached approximately 1890.0 ELU/mL, with 100% achieving concentrations ≥ 150 ELU/mL and 94.9% demonstrating at least a four-fold increase. In two years, however, the geometric mean concentration had declined to 348.9 ELU/mL, with 64.0% retaining concentrations ≥ 150 ELU/mL. In children aged 2–<5 years, the initial response was substantially weaker, with a six-week geometric mean concentration of 832.2 ELU/mL, 85.7% achieving ≥150 ELU/mL, and 70.7% demonstrating a four-fold increase. The decline was particularly pronounced during follow-up: at two years, the geometric mean concentration was only 133.4 ELU/mL, with 38.1% retaining concentrations ≥ 150 ELU/mL and only 25.0% maintaining a four-fold increase [47]. These age-related differences are consistent with the reduced capacity of young children to generate robust and durable responses to T-cell-independent polysaccharide antigens.

The clinical relevance of these immunological findings is reflected in the limitations of ViPS for young children and long-term population protection. Although ViPS can provide meaningful short-term protection following a single injection, its limited induction of T-cell-dependent memory and progressive antibody waning necessitate periodic revaccination, generally at approximately two- to three-year intervals in populations where continued protection is required [52]. Moreover, the relatively poor immunogenicity of unconjugated polysaccharide vaccines in children below two years of age represents a major limitation because this age group may experience substantial typhoid exposure [51]. Community-wide vaccination programs can nevertheless generate indirect population-level benefits by reducing transmission and exposure among unvaccinated individuals. However, the limitations of T-cell-independent immunity, particularly in young children and with respect to long-term immune memory, provided a major immunological rationale for the subsequent development of Vi conjugate vaccines (TCVs), in which the Vi polysaccharide is linked to a protein carrier to recruit T-helper-cell-dependent immune responses.

### 2.2. Typhoid Conjugate Vaccines (TCVs)

To overcome the limited immunological memory and age-related limitations of unconjugated Vi polysaccharide vaccines, typhoid conjugate vaccines (TCVs) were developed by covalently linking the *Salmonella enterica* serovar Typhi (*S.* Typhi) Vi capsular polysaccharide to a protein carrier. This conjugation converts the predominantly T-cell-independent response induced by Vi polysaccharide into a T-cell-dependent immune response, thereby improving immunogenicity and the development of immunological memory [52,53]. Following administration, antigen-presenting cells process the protein carrier component and present carrier-derived peptides through major histocompatibility complex (MHC) class II molecules to CD4+ T-helper cells [54,55]. At the same time, Vi-specific B cells recognize the polysaccharide component through their B-cell receptors. Interaction between activated T-helper cells and Vi-specific B cells promotes B-cell proliferation, class switching, affinity maturation, and the formation of memory B cells and long-lived plasma cells [55]. This mechanism results in stronger and more persistent anti-Vi antibody responses than those generally generated by unconjugated ViPS vaccines and permits effective vaccination during infancy and early childhood.

Several TCV formulations using different carrier proteins have been evaluated clinically, including Vi-tetanus toxoid (Vi-TT) [56], Vi-CRM_197_ [57], and Vi-diphtheria toxoid (Vi-DT) [58]. Vi-TT, exemplified by Typbar-TCV^®^, contains purified Vi polysaccharide conjugated to tetanus toxoid and is administered as a single intramuscular dose [56]. Typbar-TCV^®^ was prequalified by the World Health Organization (WHO) in 2017 and has been evaluated in children and adults, including children from six months of age. Clinical trials conducted in different endemic settings have demonstrated substantial protection against blood culture-confirmed typhoid fever. Across phase III studies, reported efficacy has been approximately 79–85%, including trials conducted in Nepal, Malawi, and Indonesia [59,60,61]. Effectiveness has also been demonstrated during outbreaks involving extensively drug-resistant *S.* Typhi, providing evidence that TCV vaccination can reduce disease even in settings where treatment options are increasingly constrained by antimicrobial resistance [62].

The clinical performance of TCVs is supported by their ability to generate strong and sustained antibody responses. Unlike ViPS, which primarily stimulates a T-cell-independent response with limited immunological memory, TCVs recruit carrier-protein-specific T-helper cells and consequently support the formation of memory B cells and long-lived antibody-producing plasma cells [53,63]. This immunological mechanism contributes to the longer duration of protection observed following TCV vaccination. Depending on the formulation and available follow-up data, protection has been demonstrated for several years, with Vi-TT studies supporting protection extending to approximately seven years [32], whereas Vi-CRM_197_ formulations have demonstrated sustained immune responses following vaccination; available evidence suggests that TCVs can provide protection over several years; however, the duration of protection is formulation- and study-dependent [57,64,65,66,67]. The duration and durability of protection remain dependent on the vaccine formulation, age at vaccination, and population studied, and further long-term follow-up is needed to determine the optimal need and timing of booster doses.

The improved immunological profile of TCVs is particularly important for young children. Whereas unconjugated ViPS vaccines have limited immunogenicity in children below two years of age, TCVs can generate effective immune responses from six months of age [61]. This characteristic led to the WHO recommendation for TCV introduction into routine immunization programs in typhoid-endemic and high-risk settings, with catch-up vaccination campaigns extending protection to older children and adolescents where appropriate [68]. TCVs can also be administered alongside other routine childhood vaccines without clinically important interference, facilitating their integration into existing immunization schedules. Their single-dose administration further reduces some of the adherence and program-complexity challenges associated with multi-dose vaccines such as Ty21a.

Other TCV formulations have demonstrated comparable immunological performance. Vi-CRM_197_, represented by TYPHIBEV^®^, consists of Vi polysaccharide conjugated to CRM_197_, a non-toxic mutant of diphtheria toxin. Clinical studies have demonstrated safety and immunogenicity that are comparable or non-inferior to Vi-TT, and the vaccine has been WHO prequalified [69]. Vi-DT, in which Vi polysaccharide is conjugated to diphtheria toxoid, has similarly demonstrated non-inferior safety and immunogenicity compared with Vi-TT in phase III studies involving individuals from six months to 45 years of age [64]. An additional candidate, Vi-rEPA, in which Vi polysaccharide is conjugated to recombinant exoprotein A from *Pseudomonas aeruginosa*, demonstrated approximately 89–91% protective efficacy in children aged 2–5 years in clinical studies, although this formulation has not been commercialized [70,71]. These findings collectively demonstrate that protein conjugation can substantially improve the immunological performance of Vi-based vaccination, although clinical efficacy and duration of protection cannot be assumed to be identical across different carrier proteins and formulations.

TCVs have generally demonstrated favorable safety profiles in infants, children, adolescents, and adults. Reported adverse events are predominantly mild and include transient fever and local injection-site reactions such as pain, erythema, and swelling [72]. Nevertheless, TCVs retain important biological and programmatic limitations. Their antigenic target is primarily the Vi capsule of *S.* Typhi; therefore, they are not expected to provide protection against *S.* Paratyphi A, which generally lacks the Vi capsule, or against Vi-negative *S.* Typhi strains. Consequently, increasing recognition of paratyphoid fever highlights the need for future vaccines capable of providing broader protection against the major causes of enteric fever. Furthermore, because currently licensed TCVs are administered parenterally, they do not specifically reproduce the mucosal immune responses associated with orally administered vaccines such as Ty21a. Breakthrough infections can therefore occur, particularly in settings with intense exposure, although vaccination can substantially reduce the risk of clinical typhoid disease.

Despite their improved immunological and clinical performance, TCV implementation also faces practical challenges. The vaccines generally require cold-chain storage at approximately 2–8 °C [73], making reliable refrigeration, transportation, and vaccine-storage infrastructure necessary for deployment. Upfront procurement costs, health-system capacity, community engagement, and uneven access to vaccination services can also influence program uptake, particularly in settings with constrained health budgets or limited access to international vaccine financing mechanisms [74,75,76,77]. Thus, although TCVs represent a major advance over Ty21a and ViPS in terms of age eligibility, immunological memory, durability, and clinical efficacy, their deployment remains dependent on conventional vaccine manufacturing and distribution systems. A comprehensive overview of key clinical trials evaluating licensed typhoid vaccines, including Ty21a, ViPS, TCV, and Vi-rEPA, detailing trial phases, routes of administration, age indications, protective efficacy or effectiveness, and safety outcomes, is summarized in Table 1.

**Figure 1:**
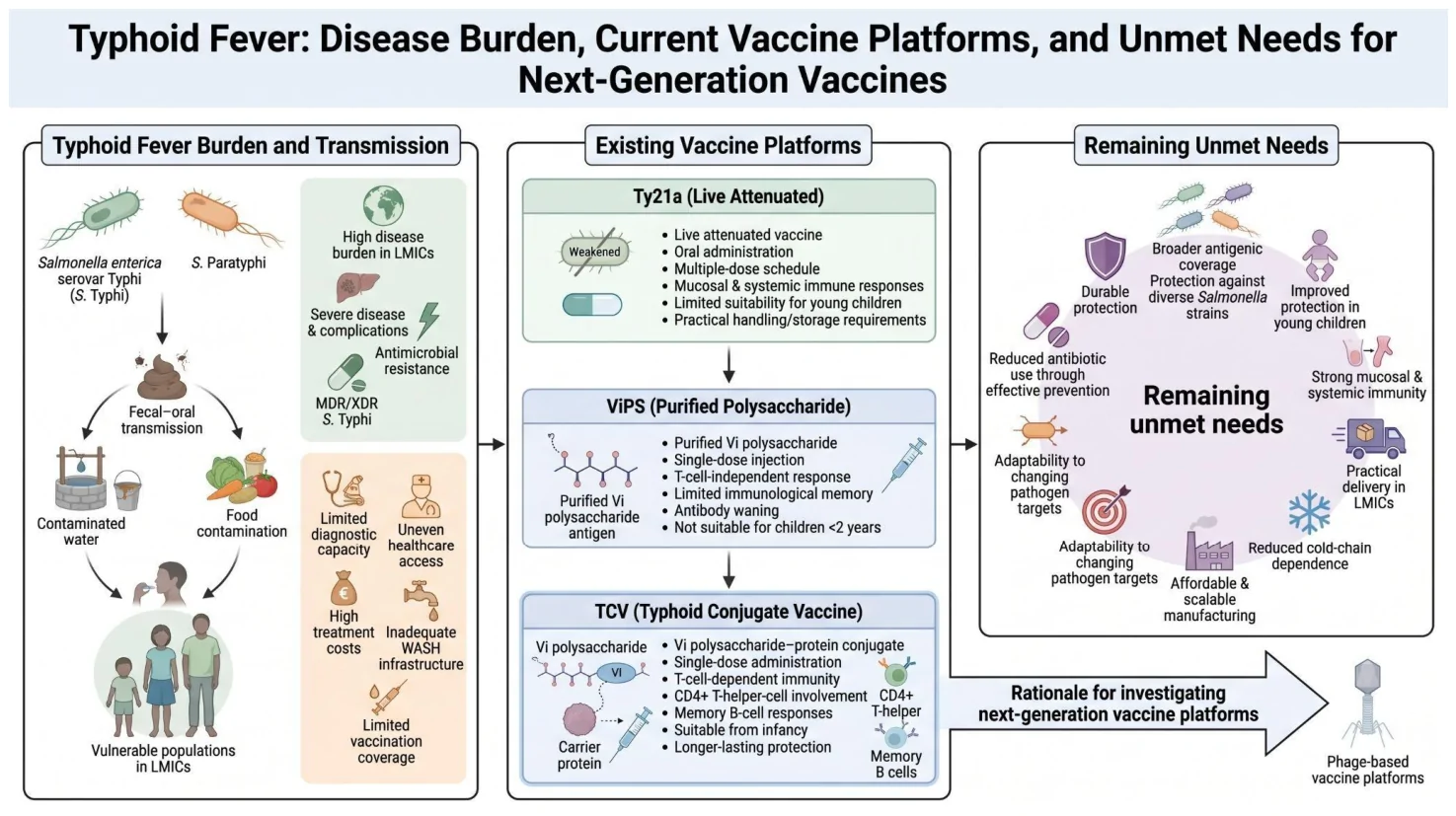
Typhoid fever disease burden, limitations of existing vaccine platforms, and key unmet needs driving next-generation solutions. Schematic representation of the global epidemiological drivers of enteric fever (*Salmonella enterica* serovars Typhi and Paratyphi), comparing currently available typhoid vaccines against key clinical and logistical requirements. Typhoid Fever Burden and Transmission: Transmission occurs predominantly via the fecal–oral route through contaminated water and food supplies, disproportionately impacting vulnerable populations in low- and middle-income countries (LMICs). The overall disease burden is exacerbated by severe clinical complications, the rise in multidrug-resistant (MDR) and extensively drug-resistant (XDR) *S.* Typhi strains, limited diagnostic capacity, uneven healthcare access, high treatment costs, inadequate water, sanitation, and hygiene (WASH) infrastructure, and low immunization coverage. Existing Vaccine Platforms: Evaluation of current options, including live-attenuated Ty21a (oral, multi-dose, induces mucosal/systemic responses, but limited in young children and requiring practical handling), unconjugated Vi capsular polysaccharide (ViPS; injectable single-dose, T-cell-independent, prone to antibody waning, unsuitable for children < 2 years), and Vi typhoid conjugate vaccines (TCVs; protein-conjugated, T-cell-dependent, suitable from infancy, providing long-lasting memory). Remaining Unmet Needs: Critical targets for novel candidates, including broader antigenic coverage across diverse *Salmonella* strains, improved protection in young children, strong dual mucosal/systemic immunity, reduced cold-chain dependence, affordable and scalable biomanufacturing, rapid adaptability to novel pathogen variants, and reduced reliance on antibiotics through effective prevention. Together, these gaps provide a clear rationale for investigating phage-based delivery systems as next-generation vaccine platforms. In the schematic, solid arrows indicate directional relationships or progression between the depicted processes and vaccine-development considerations, whereas dashed connectors within the TCV panel indicate the association of the Vi polysaccharide with the carrier protein and its incorporation into the conjugate vaccine formulation (Figure created with BioRender.com).

Overall, TCVs currently represent the most clinically advanced and widely adopted typhoid vaccine platform, with evidence spanning immunological studies, randomized clinical trials, field effectiveness, and programmatic implementation. Their ability to induce T-cell-dependent immunity and durable antibody responses while protecting children from six months of age addresses several major limitations of earlier vaccines. However, their restricted antigenic coverage, lack of direct mucosal immunization, continued dependence on cold-chain systems, and remaining implementation barriers provide important considerations when evaluating alternative next-generation platforms, including bacteriophage-based vaccine technologies. The epidemiological burden of typhoid fever, the clinical features and limitations of licensed vaccine platforms, and the key unmet needs driving the rationale for phage-based delivery systems are summarized in Figure 1.

## 3. Phage-Based Vaccine Platforms for Typhoid Fever

Bacteriophages (phages) are viruses that specifically infect bacteria and do not replicate in mammalian cells [80], characteristics that have supported their development as platforms for antigen presentation and nucleic-acid delivery. In vaccine research, phages can be genetically or chemically modified to display antigenic peptides or proteins on their capsid surfaces, to carry nucleic-acid sequences encoding vaccine antigens, or to combine both approaches within a single construct. Their repetitive capsid architecture provides a particulate scaffold for multivalent antigen presentation, while phage-associated proteins and nucleic acids can interact with innate immune-sensing pathways and contribute to immunostimulatory activity [81,82,83]. However, the magnitude and quality of these responses depend on phage species, capsid composition, antigen design, purification, administration route, and host. Thus, the immunological properties of phages should be considered construct-specific rather than universal characteristics of the platform.

The highly organized and repetitive capsid structures of filamentous phages can permit high-density presentation of antigenic determinants and facilitate interactions with antigen-presenting cells. Surface display may favor antibody responses against exposed epitopes, whereas phage-associated DNA constructs can potentially provide endogenous antigen expression and promote cellular immune responses [84]. Hybrid designs may therefore combine direct antigen presentation with intracellular antigen production. Nevertheless, these potential advantages have not been demonstrated to provide superior protection against *Salmonella enterica* serovar Typhi (*S.* Typhi) in humans. In particular, broad protection against *S.* Typhi and *S.* Paratyphi has not been established for phage-based vaccines and should therefore be regarded as a development objective rather than an established property.

Importantly, the evidence base for phage-based vaccination against typhoid fever remains at the conceptual and platform-development stage. To date, no bacteriophage-based vaccine specifically designed to prevent typhoid fever has been evaluated in a preclinical efficacy study or human clinical trial. Consequently, evidence from phage-based vaccine studies targeting unrelated pathogens should be interpreted primarily as platform-level proof of concept rather than as direct evidence of protective efficacy against typhoid fever. Where available, greater emphasis should therefore be placed on studies involving *Salmonella* species, *S.* Typhi-relevant antigens, and closely related particulate vaccine technologies that can inform antigen selection, delivery, immunogenicity, and platform design.

One relevant example is the use of the bacteriophage-derived Qβ platform in experimental *Salmonella* vaccination. Qβ has been investigated as a particulate carrier for synthetic *Salmonella* Paratyphi A O-polysaccharide antigens. In a murine model, the resulting conjugate induced substantial antigen-specific IgG responses, and passive transfer of immune sera provided protection against lethal *S.* Paratyphi A challenge [85]. Although this approach represents preclinical rather than clinical evidence and Qβ-based constructs may function primarily as bacteriophage-derived virus-like particles rather than conventional replicating phage vectors, the findings are directly relevant to enteric *Salmonella* vaccine development. They demonstrate that bacteriophage-derived particulate scaffolds can be engineered to present *Salmonella*-specific carbohydrate antigens and generate antibodies with protective activity in an animal model.

Additional *S.* Typhi-specific evidence comes from recent particulate vaccine research outside the bacteriophage field. Multivalent particulate vaccine strategies have also been explored using immunogenic epitopes derived from multiple *S.* Typhi antigens, including outer membrane proteins such as OmpC and OmpF and flagellin [86]. Such approaches illustrate the potential value of combining conserved or immunogenic antigenic regions to broaden immune recognition against *S.* Typhi. Structural characterization confirmed successful assembly of the antigen–AP205 particle complexes, providing a recent proof of concept for multivalent presentation of *S.* Typhi antigens on an organized nanoscale particulate scaffold [87]. Although AP205 is a virus-like particle (VLP) rather than a bacteriophage, the study provides *S.* Typhi-specific evidence that multiple protein epitopes can be organized and presented on a particulate platform. This finding is not direct evidence for phage-based vaccine efficacy but may inform the selection and design of future phage-display constructs targeting *S.* Typhi.

These studies highlight an important issue that has received insufficient attention in the development of phage-based typhoid vaccines; antigen selection. Unlike pathogens for which a single dominant protective antigen may be sufficient, typhoid fever involves a systemic infection in which bacterial surface structures, virulence-associated proteins, and intracellular antigens may contribute differently to protective immunity. Candidate antigens for phage display or nucleic-acid delivery could therefore include the Vi capsular polysaccharide or Vi-associated epitopes, outer membrane proteins such as OmpC and OmpF, flagellin, and conserved virulence-associated proteins. The choice should be guided by evidence of surface accessibility, conservation among circulating *S.* Typhi strains, expression during infection, ability to induce functional antibodies or T-cell responses, and potential contribution to protection. Importantly, antigen selection should also consider *S.* Paratyphi A because Vi-based strategies alone cannot provide comprehensive protection against paratyphoid fever [31,88]. Multivalent constructs incorporating conserved antigens shared across clinically important *Salmonella* serovars may therefore provide a rational direction for future development, although their ability to generate cross-protective immunity remains to be experimentally established.

### 3.1. Antigen Selection Strategies for Phage-Based Typhoid Vaccines

The selection of an appropriate antigen is arguably the most important determinant of whether a phage-based platform can generate meaningful protection against typhoid fever. *S.* Typhi is an intracellular pathogen with a complex disease process involving intestinal invasion, systemic dissemination, survival within phagocytic cells, and persistence in host tissues [89]. Consequently, an effective vaccine may need to generate more than circulating antibodies against a single surface antigen. Antigen selection should consider antigen abundance, conservation across circulating *S.* Typhi strains, accessibility to the immune system, contribution to bacterial virulence, ability to induce functional antibodies, and capacity to stimulate cellular immune responses.

#### 3.1.1. Vi Capsular Polysaccharide

The Vi capsular polysaccharide is an obvious candidate because it is an established protective antigen and is already exploited by licensed Vi polysaccharide and typhoid conjugate vaccines [46,56,57,58]. Incorporating Vi-associated epitopes into a phage platform could therefore provide a direct conceptual link between established typhoid vaccinology and emerging phage technology. A phage displaying Vi-derived epitopes or Vi mimotopes could potentially exploit the repetitive architecture of the capsid to generate high-density antigen presentation.

However, the Vi antigen also presents important limitations. Vi is predominantly associated with *S.* Typhi and is absent from *S.* Paratyphi A [90], meaning that Vi-focused vaccination would not necessarily provide broad protection against paratyphoid fever. Furthermore, the immune response to unconjugated polysaccharide antigens is predominantly T-cell independent and therefore less effective in young children. A phage platform would need to demonstrate that its particulate presentation or incorporation of appropriate carrier sequences can overcome this limitation rather than simply reproduce the immunological characteristics of conventional Vi polysaccharide vaccination.

A potentially more sophisticated approach would therefore involve the display of defined Vi epitopes or mimotopes on a phage scaffold together with additional protein antigens capable of inducing T-cell responses. Such multicomponent designs could preserve the established protective relevance of Vi while addressing the limited antigenic breadth of Vi-only vaccines.

#### 3.1.2. Outer Membrane Proteins

Outer membrane proteins (OMPs) represent another important antigen class. Proteins such as OmpA, OmpC, OmpD, OmpF and other conserved outer-membrane components have attracted interest because they are exposed to the host immune system and may contain epitopes recognized by antibodies or T cells [91,92]. Unlike Vi, several OMPs are protein antigens and may therefore provide opportunities for both humoral and cellular immune stimulation.

Phage display is particularly attractive for OMP-derived epitopes because relatively small antigenic regions can potentially be genetically fused to capsid proteins. This approach may allow the presentation of defined protective epitopes while avoiding the expression of entire membrane proteins that could interfere with phage assembly or antigen folding. However, antigenicity does not necessarily equate to protection. OMP candidates should therefore be evaluated for conservation among circulating *S.* Typhi isolates, surface accessibility during infection, functional antibody activity, and their ability to contribute to protection in appropriate animal models.

#### 3.1.3. Flagellin

Flagellin is another candidate of interest because it is the major structural component of the bacterial flagellum and is highly immunogenic [93,94,95]. Flagellin can stimulate innate immune pathways, particularly through Toll-like receptor 5 (TLR5), while also providing antigen-specific adaptive immune targets [93]. Its combination of antigenic and innate immunostimulatory properties makes flagellin-containing constructs potentially attractive for phage-based vaccine development.

Nevertheless, flagellin-based strategies require careful consideration of antigenic variability and immune focusing. A highly immunogenic antigen may dominate the response without necessarily generating the most protective immunity. Furthermore, excessive innate stimulation could potentially increase reactogenicity. For phage vaccines, the density and orientation of flagellin-derived epitopes on the capsid would therefore need to be optimized rather than assuming that maximal display automatically produces maximal protection.

#### 3.1.4. Conserved Salmonella Proteins

Conserved intracellular and virulence-associated proteins provide an additional opportunity for developing broader vaccine coverage [96]. Proteins involved in invasion, intracellular survival, metabolism, secretion systems, and stress responses may contain conserved T-cell epitopes that are less affected by surface antigenic variation [97]. These antigens could be particularly valuable in DNA or hybrid phage systems in which intracellular antigen expression is intended to stimulate cellular immunity.

A rational strategy would therefore move away from selecting antigens solely according to abundance or surface exposure and instead combine complementary antigen classes. For example, a candidate could incorporate a Vi-derived component to promote antibody-mediated recognition, an OMP or flagellin-derived component to enhance surface-directed immunity, and conserved intracellular antigens to promote T-cell responses against infected cells. Such a strategy could theoretically address several stages of *S.* Typhi infection, although the protective value of these combinations must be demonstrated experimentally.

Overall, antigen selection for a typhoid phage vaccine should be guided by a combination of comparative genomics, immunoproteomics, structural analysis, epitope mapping, and experimental immunogenicity studies. The objective should not simply be to identify antigens that can be displayed on a phage but to identify combinations that generate functional immunity against the biological stages responsible for typhoid disease.

### 3.2. Phage Vaccine Architectures and Salmonella-Specific Evidence

**Figure 2:**
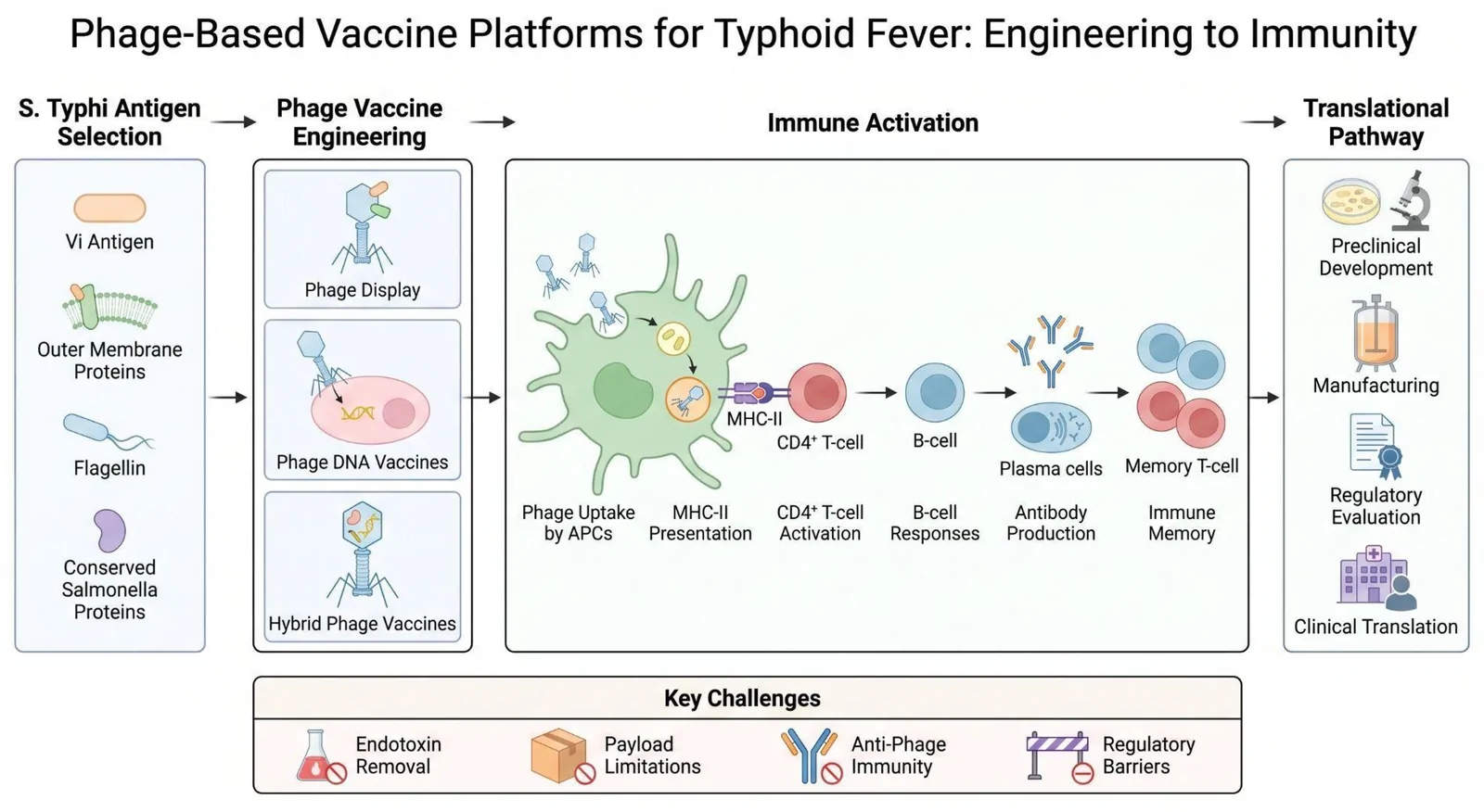
Translational continuum of bacteriophage-based typhoid vaccines: From target selection to clinical implementation. Schematic depiction of the four sequential stages in phage vaccine development against *Salmonella* Typhi: (1) *S.* Typhi Antigen Selection: Identification of key protective targets, including the Vi capsular polysaccharide, outer membrane proteins (OMPs), flagellin, and conserved Salmonella proteins. (2) Phage Vaccine Engineering: Formulation into distinct delivery platforms, including phage display (surface presentation of peptide/protein antigens on capsid proteins), phage DNA vaccines (delivery of eukaryotic expression cassettes encapsidated within phage particles), and hybrid phage platforms (combining surface display with gene delivery). (3) Immune Activation: Internalization of engineered phage constructs by antigen-presenting cells (APCs), followed by intracellular processing and MHC class II antigen presentation. Engagement of CD4+ helper T cells drives B-cell differentiation into antibody-secreting plasma cells and the establishment of long-term cellular immune memory. (4) Translational Pathway: Key developmental milestones spanning preclinical evaluation, large-scale bioreactor biomanufacturing under Good Manufacturing Practice (GMP) conditions, regulatory approval, and clinical translation. Key Translational Challenges: Major obstacles impeding clinical transition, including downstream endotoxin removal, viral packaging/payload limitations, rapid vector clearance via anti-phage immunity, and complex regulatory frameworks for engineered biological entities (Figure created with BioRender.com).

Phage-based vaccines can broadly be divided into three architectures: phage display vaccines, phage DNA vaccines, and hybrid phage vaccines. These approaches differ in how antigen is presented, where antigen expression occurs, and which components of the immune system are expected to dominate the response. The translational pipeline of phage-based typhoid vaccines, spanning *Salmonella Typhi* antigen selection, engineering modalities, APC uptake and immune activation pathways, translational milestones, and key developmental hurdles, is illustrated in Figure 2.

#### 3.2.1. Phage Display Vaccines

Phage display vaccines exploit the ability of filamentous phages to present foreign peptides or proteins on exposed capsid proteins [98,99]. Antigen display can create a repetitive particulate array that facilitates B-cell receptor cross-linking and antigen uptake by antigen-presenting cells, potentially enhancing humoral and cellular immune responses. The density and orientation of the displayed antigen can be manipulated through the choice of phage coat protein, insertion site, and display system [100]. This provides an opportunity to present multiple epitopes within the same particle rather than relying on a single soluble antigen.

For typhoid vaccine development, however, the central challenge is identifying antigens that are both appropriately displayed and biologically relevant during *S.* Typhi infection. OmpC, OmpF, flagellin, and selected Vi-associated epitopes represent potential candidates because they are associated with bacterial surface structures or virulence and have been investigated in *Salmonella* vaccine research [91,92]. The recent demonstration of OmpC, OmpF, and flagellin-derived epitopes on AP205 VLPs [87] provides a useful *S.* Typhi-specific precedent for combining several protein antigens on a particulate scaffold. Translation of this approach to bacteriophage particles would require optimization of epitope accessibility, structural stability, antigen density, and preservation of relevant conformational epitopes.

The Qβ/Salmonella studies provide another relevant precedent [85]. Presentation of *S.* Paratyphi A *O*-polysaccharide on a bacteriophage-derived particulate scaffold generated strong antigen-specific IgG responses and protective activity following passive serum transfer in mice [85]. This finding is particularly important because it demonstrates that bacteriophage-derived particles can present a carbohydrate antigen relevant to enteric salmonellosis, rather than relying exclusively on unrelated pathogen models. Nevertheless, the study does not establish protection against *S.* Typhi, and the immune mechanisms generated against *O*-polysaccharide cannot automatically be extrapolated to Vi or protein antigens.

Phage display also presents technical challenges. Foreign peptide insertion can alter capsid assembly, folding, particle stability, or infectivity, while excessive antigen density may not necessarily translate into improved immunity [80,98]. These problems can be addressed through rational selection of insertion sites, use of flexible linkers, screening of multiple display densities, and structural characterization of the resulting particles. Antigen engineering should also favor conserved epitopes that retain their native immunological properties rather than simply maximizing the amount of displayed material. Comparative studies of different display architectures and doses will therefore be required before claims of enhanced immunogenicity can be justified.

#### 3.2.2. Phage DNA Vaccines

Phage DNA vaccines use engineered phage particles to package and deliver expression cassettes encoding vaccine antigens [101]. Following delivery, the antigen-encoding sequence must enter an appropriate host–cell compartment and undergo transcription and translation, allowing endogenous antigen production and potentially enhancing presentation through MHC class I and class II pathways [102]. This approach could theoretically complement the predominantly antibody-focused response generated by surface-displayed antigens.

Evidence from anthrax, plague, HIV, and other models has demonstrated that phage-associated DNA can induce antigen-specific immune responses [20,103,104]. However, these studies should be regarded as platform feasibility studies rather than evidence for typhoid protection, because antigen processing, tissue tropism, cellular immune requirements, and protective mechanisms differ substantially between these pathogens and *S.* Typhi. For typhoid fever, the more relevant question is whether phage DNA delivery can efficiently express appropriately selected *S.* Typhi antigens and generate immune responses capable of controlling both extracellular and intracellular stages of infection.

Candidate genes could include selected OMPs, flagellin, and conserved virulence-associated proteins. However, selection should be based on evidence that the encoded antigen is expressed during infection and can generate functional antibody or T-cell responses. Combining conserved antigens from *S.* Typhi and *S.* Paratyphi could potentially broaden coverage, but such designs remain hypothetical until evaluated experimentally.

Several technical barriers must also be addressed. Endotoxin contamination is particularly important because phages are commonly propagated in Gram-negative bacterial hosts, and residual lipopolysaccharide can produce strong innate inflammatory responses that confound vaccine-specific effects and compromise safety [105,106]. Potential mitigation strategies include optimized host strains with reduced endotoxin production, rigorous downstream purification, chromatographic separation, membrane-based purification, and validated endotoxin-removal procedures [107,108,109]. Endotoxin levels should be measured quantitatively in every vaccine preparation rather than assuming that purification has eliminated the problem.

A second limitation is low mammalian-cell delivery and expression efficiency. Phages naturally evolved to interact with bacterial cells and therefore do not possess the same cellular entry and trafficking mechanisms as mammalian viral vectors [110]. This limitation can be addressed experimentally through optimization of the phage capsid, incorporation of mammalian cell-targeting ligands, optimization of promoter and expression-cassette design, and evaluation of intracellular trafficking and antigen expression in relevant antigen-presenting cells. Such modifications must be assessed carefully because increasing cellular targeting may also alter particle biodistribution, immunogenicity, or safety.

Thus, phage DNA vaccines should currently be regarded as an experimental delivery strategy rather than an established alternative to conventional DNA or viral-vector vaccines. Demonstration of efficient antigen expression and protective immunity against *S.* Typhi in appropriate animal models will be necessary before clinical translation can be considered.

#### 3.2.3. Hybrid Phage Vaccines

Hybrid phage vaccines combine surface antigen display with delivery of antigen-encoding nucleic acids within the same particle [111]. The proposed rationale is that surface-displayed antigens could provide immediate exposure to B cells and antigen-presenting cells, while intracellular expression of encoded antigens could promote additional cellular immune responses. In principle, this dual presentation could address the need for both antibody-mediated and cellular immunity in systemic *Salmonella* infection.

However, hybrid phage vaccines should not currently be considered as the superior phage platform, because there is insufficient comparative evidence to establish superiority, particularly for typhoid fever. The simultaneous presentation of two antigenic modalities could produce synergistic immune activation, but it could also generate immunological interference [112,113]. For example, antibodies generated against the surface-displayed antigen may bind the phage particle during subsequent exposure and accelerate its clearance before sufficient delivery of the DNA cargo occurs. Conversely, strong cellular or antibody responses generated against the DNA-expressed antigen may alter subsequent responses to the surface antigen. The density, immunodominance, and antigenic relationship between the two components could therefore determine whether the combination produces synergy or competition.

This possibility of carrier-directed neutralization is particularly important for repeated vaccination [114]. If antibodies against the surface antigen also recognize the phage particle or otherwise promote rapid particle uptake and clearance, subsequent doses may have reduced access to cells capable of expressing the DNA-encoded antigen. Hybrid designs should therefore evaluate anti-phage antibody development, particle clearance, tissue distribution, DNA delivery, and antigen expression after both primary and booster administration. Strategies such as altering display density, using less immunodominant carrier surfaces, modifying administration schedules, or employing heterologous phage backbones could potentially reduce this problem, but these approaches require experimental validation.

For *S.* Typhi, a rational hybrid design could combine a surface-exposed antigen such as a selected Vi-associated or outer-membrane epitope with DNA encoding one or more conserved protein antigens such as OmpC, OmpF, or flagellin. Such a design could theoretically provide complementary antibody and cellular responses. However, the appropriate combination should be determined experimentally rather than assumed to be synergistic. In particular, studies should compare display-only, DNA-only, and hybrid constructs using identical antigen doses and assess antibody titers, functional bactericidal activity, CD4+ and CD8+ T-cell responses, memory responses, bacterial clearance, and protection following *S.* Typhi challenge.

Evidence from hybrid phage vaccine studies involving *Candida albicans* and influenza demonstrates that combining antigen-display and nucleic-acid delivery strategies can alter immune responses compared with single-modality approaches [115,116]. Nevertheless, because these pathogens differ substantially from *S.* Typhi in their tissue tropism, antigenic targets, and protective immune mechanisms, these studies should be interpreted as platform-level evidence rather than evidence of efficacy against typhoid fever. The next critical step is therefore direct evaluation of hybrid phage constructs carrying carefully selected *S.* Typhi antigens in relevant animal models.

#### 3.2.4. Comparative Features of Major Phage Vaccine Platforms

The major phage platforms used in vaccine development differ substantially in capsid architecture, genome type, antigen-display strategy, payload capacity, production characteristics, and potential suitability for mucosal administration [80,117]. Among the platforms discussed in this review, T4 and M13 represent structurally distinct phage-based display systems, whereas Qβ and AP205 are commonly developed as bacteriophage-derived virus-like particles (VLPs) that retain the particulate architecture of their parental phages but generally do not contain replication-competent phage genomes in the final vaccine formulation [85,87]. These differences are important when selecting a platform for typhoid vaccine development because antigen size, display density, particle stability, manufacturing requirements, and intended route of administration can all influence vaccine performance.

T4 provides a comparatively large and structurally robust capsid that can accommodate high-density display through the non-essential Hoc and Soc capsid proteins. The T4 architecture is therefore particularly attractive when larger proteins or multiple antigenic components are required, and recombinant T4 particles have demonstrated the capacity to maintain structural integrity while presenting complex antigens [22,23,24]. T4 has also been investigated for mucosal and needle-free vaccine administration, including studies demonstrating induction of mucosal immune responses following respiratory or other non-parenteral delivery [21,27]. Its large particle size and multiple independent display sites provide considerable engineering flexibility, although increasing antigen size or density can still influence particle assembly, stability, and production yield.

M13 is a filamentous, non-lytic phage with a long and flexible morphology. Its major coat protein pVIII occurs at approximately 2750 copies per particle and is therefore particularly useful for high-density display of short peptides, whereas pIII is commonly used when larger peptide or protein fragments are required [98,99]. M13 has a well-developed phage-display technology base and can be propagated efficiently in *Escherichia coli*, contributing to its extensive use in vaccine research and large-scale experimental applications. However, the secretion and assembly requirements associated with its filamentous coat proteins can constrain the display of large or structurally complex proteins. M13 has nevertheless demonstrated practical translational potential, including field evaluation of an M13-based cysticercosis vaccine in pigs [25,26]. Its long, flexible structure and established display technology may be advantageous for epitope-focused vaccines, although these features do not necessarily confer an advantage over the larger T4 scaffold for complex multivalent protein antigens.

Qβ and AP205 differ from T4 and M13 in that they are small positive-sense RNA phages that are commonly exploited as self-assembling VLP platforms rather than as conventional replicating phage vectors. Qβ forms approximately 28 nm particles composed of 180 coat-protein subunits and can present peptide epitopes through genetic fusion or larger antigens through chemical conjugation. Qβ-based VLPs have generated strong humoral responses and have progressed further in translational development than many conventional phage-display systems, including preclinical and clinical evaluation of several vaccine applications [118]. AP205 forms a similarly compact approximately 27–30 nm particle containing 180 coat-protein subunits and permits genetic fusion of relatively short peptides to its coat protein. Its repetitive surface architecture and packaged RNA-associated innate immune activity can contribute to immunogenicity, while the SpyTag/SpyCatcher system provides an additional strategy for modular antigen attachment [16]. Importantly, AP205-based presentation of OmpC, OmpF, and flagellin-derived *S.* Typhi epitopes provide a pathogen-specific precedent for particulate and multivalent antigen presentation [86,87]. To guide platform selection for typhoid vaccine engineering, the structural architectures, payload capacities, immunogenicity profiles, manufacturing systems, physical stability, and potential suitability for oral or mucosal administration across major phage-derived platforms (T4, M13, Qβ, and AP205) are comparative summarized in Table 2.

The four platforms therefore represent different engineering trade-offs rather than a simple hierarchy of performance. T4 is particularly attractive for large proteins, multiple antigens, and combined display strategies; M13 is well suited to high-density peptide presentation and established phage-display workflows; Qβ provides a compact, highly repetitive VLP scaffold with substantial experience in conjugate vaccine development; and AP205 offers a similarly compact particulate scaffold with flexible antigen attachment and demonstrated relevance to *S.* Typhi antigen presentation. Their relative suitability for oral vaccination is also not determined solely by particle architecture. Successful oral delivery requires preservation of particle integrity through the gastrointestinal environment, resistance to proteolytic and chemical degradation, appropriate interaction with intestinal immune tissues, and efficient uptake by mucosal antigen-presenting cells. Consequently, direct comparative studies under identical oral dosing conditions are required before one platform can be considered superior for typhoid vaccination.

#### 3.2.5. Oral and Mucosal Delivery of Phage-Based Vaccines

The gastrointestinal tract (GIT) represents an important target for typhoid vaccination because *S.* Typhi is acquired predominantly through the ingestion of contaminated food or water and initially encounters the intestinal mucosal surface. Consequently, vaccine strategies capable of generating immunity at the intestinal portal of entry may provide an additional layer of protection beyond systemic antibody responses. The currently licensed oral Ty21a vaccine illustrates this principle (oral administration permits interaction with intestinal immune tissues and induces both mucosal and systemic immune responses), including antigen-specific cellular responses within the intestinal mucosa [38,39]. In contrast, most currently available typhoid conjugate vaccines are administered parenterally and therefore do not specifically reproduce the mucosal immune response associated with oral vaccination.

Phage particles are potentially attractive for mucosal delivery because their particulate architecture can facilitate antigen uptake and presentation by mucosal antigen-presenting cells. Following oral administration, an engineered phage particle would need to survive the acidic gastric environment and digestive enzymes sufficiently to reach the small intestine while retaining the structural integrity and antigenic properties required for immune recognition. At the intestinal surface, interaction with the mucus layer, epithelial cells, M cells, dendritic cells, and other components of gut-associated lymphoid tissue could influence particle retention, uptake, antigen processing, and the resulting immune response. The desired outcome would include local mucosal immunity, particularly secretory IgA (SIgA), together with systemic antibody and T-cell responses capable of limiting dissemination following invasion. However, these outcomes cannot be assumed solely from the particulate nature of phages and require direct experimental demonstration.

Different phage architectures may behave differently following oral administration. Large particles such as T4 provide extensive surface-engineering capacity and have demonstrated mucosal immunogenicity following non-parenteral administration in other disease models [21,27]. Filamentous M13 particles have also been investigated in needle-free and mucosal vaccine approaches [119], while compact Qβ and AP205 VLPs provide highly ordered particulate scaffolds that may be suitable for oral antigen delivery. Nevertheless, gastrointestinal stability and mucosal uptake are formulation-dependent properties. Protection of the particle from gastric degradation, optimization of antigen display density, incorporation of appropriate mucoadhesive or enteric-protective strategies, and controlled release in the intestine may therefore be necessary for effective oral administration.

For a future oral phage-based typhoid vaccine, preclinical evaluation should extend beyond serum antibody titers. Studies should assess particle integrity following simulated gastrointestinal exposure, intestinal localization and uptake, mucosal SIgA responses, antigen-specific CD4+ and CD8+ T-cell responses, systemic antibody responses, memory responses, and protection following challenges. Comparisons between oral and parenteral administration using matched antigen doses would also help determine whether the oral route provides an immunological advantage for *S.* Typhi. Importantly, the current evidence base demonstrates the feasibility of phage-mediated mucosal vaccination in other infectious disease models but does not yet establish that orally administered phage vaccines can protect against typhoid fever.

### 3.3. Phage Platforms in the Context of Emerging Particulate Vaccine Technologies

Phage-based vaccine platforms should be considered within the broader landscape of particulate vaccine technologies, including virus-like particles (VLPs), outer membrane vesicles (OMVs), and engineered nanoparticle-based vaccines. These platforms share several potentially important features, including nanoscale organization, repetitive or multivalent antigen presentation, and the capacity to enhance antigen uptake and immune recognition. However, they differ substantially in their biological composition, engineering flexibility, manufacturing requirements, and translational maturity. VLPs, for example, are non-infectious particles that lack a viral genome and can present antigens in highly ordered repetitive arrays, features that can promote strong immune responses [120]. Several VLP-based vaccines have reached clinical use [121], although production at commercial scale remains technically demanding because of challenges associated with expression systems, particle assembly, downstream processing, and product-quality control [120]. In comparison, bacteriophage platforms offer substantial genetic programmability and can be engineered for surface antigen display, nucleic-acid delivery, or both within a single particle [80]. This flexibility could permit multivalent antigen presentation and, in hybrid constructs, the combination of extracellular antigen display with endogenous antigen expression. Nevertheless, the greater engineering flexibility of phages does not establish superiority over VLPs, particularly because phage-based typhoid vaccines remain at the conceptual and platform-development stage.

OMVs represent another biologically derived particulate platform with distinctive characteristics. Because they originate from Gram-negative bacteria, OMVs naturally contain pathogen-associated molecular patterns and multiple membrane-associated components that can provide intrinsic immunostimulatory activity [122]. This self-adjuvanting property, together with their ability to present several antigens simultaneously, has contributed to the development of licensed OMV-containing vaccines and a broad preclinical and clinical research pipeline [123,124,125]. However, OMV development is complicated by the presence of lipopolysaccharide and other inflammatory components, which require careful control to balance immunostimulation with reactogenicity [126]. Additional challenges include variability in antigen composition, production yield, purification, surface loading of heterologous antigens, and long-term stability [127]. Phage platforms differ in that the identity and density of selected antigens can be more deliberately programmed through genetic engineering. At the same time, phage preparations produced in Gram-negative hosts also face the important requirement for rigorous endotoxin removal and quantitative quality control, meaning that phages do not eliminate the purification challenges associated with bacterial-derived particulate systems.

Synthetic and protein-based nanoparticles provide a further comparison because their physicochemical properties can be deliberately tuned. Particle size, antigen density, spatial organization, and surface characteristics can be optimized to influence lymphatic trafficking, antigen-presenting-cell uptake, and B-cell receptor engagement [128]. This level of control may provide considerable flexibility in antigen design and immunofocusing, but nanoparticle platforms can also encounter challenges related to formulation, particle uniformity, antigen loading, colloidal stability, reproducibility, scale-up, and long-term storage. Phage particles occupy an intermediate position between naturally occurring biological particles and engineered nanomaterials, as they possess a defined biological architecture and genetic programmability while also allowing the incorporation of selected antigens or nucleic-acid cargo. Their potential advantages therefore include modular genetic engineering, multivalent antigen display, and the possibility of combining surface presentation with genetic antigen delivery. However, these potential advantages must be weighed against phage-specific limitations, including anti-phage immunity and vector clearance, payload and capsid-assembly constraints, genetic stability, endotoxin contamination, and the limited clinical and regulatory precedent for phage-based vaccines targeting typhoid fever. A detailed comparison of core characteristics, prospective advantages, technical limitations, and translational relevance to typhoid vaccine development across phage-based platforms and competing nanoscale technologies (VLPs, OMVs, and synthetic nanoparticles) is outlined in Table 3.

Overall, the comparison indicates that no single particulate platform is intrinsically superior across all vaccine-development criteria. VLPs currently have greater clinical maturity, OMVs provide naturally immunostimulatory bacterial components and established experience in some licensed vaccines, and synthetic nanoparticles offer extensive control over particle and antigen properties. Phage platforms provide a distinctive combination of genetic programmability, multivalent display, and the potential integration of antigen presentation with nucleic-acid delivery. For typhoid fever, however, these characteristics should be regarded as development opportunities rather than demonstrated advantages. Comparative studies using equivalent antigen targets, doses, administration routes, and immunological endpoints will be required to determine whether phage-based platforms provide meaningful benefits over VLP, OMV, or nanoparticle approaches for generating protective immunity against *S.* Typhi and, where appropriate, *S.* Paratyphi.

## 4. Technical, Manufacturing, Biosafety, and Regulatory Considerations for Clinical Translation

Despite their promising prophylactic potential in preclinical models, several technical and translational hurdles must be addressed before phage-based vaccine platforms can progress to clinical use. These challenges extend beyond antigen immunogenicity and include the ability to accommodate and express heterologous payloads, manufacture purified and reproducible products at scale, maintain functional vaccine activity in the face of biological and immunological barriers, and satisfy increasingly complex biosafety and regulatory requirements. Importantly, most available evidence concerns phage engineering, phage therapy, or vaccine studies involving pathogens other than Salmonella enterica serovar Typhi. Consequently, the extent to which these limitations will affect a future phage-based typhoid vaccine remains to be experimentally established.

### 4.1. Payload and Expression Constraints

Payload capacity and heterologous gene expression represent important constraints for phage-based vaccine development. In antigen-display platforms, the size, structure, and location of a foreign antigen can affect capsid assembly, particle stability, display density, and ultimately vaccine potency [118]. Although some phages possess considerable structural tolerance for foreign proteins, this capacity is highly dependent on phage architecture and the specific capsid protein used for antigen presentation. For example, T4 phage has been engineered to display relatively large proteins through its Hoc and Soc capsid proteins, whereas filamentous phages such as M13 can be more constrained when large fusion proteins are incorporated into structural proteins involved in phage assembly and secretion [22,23,27,129,130]. Thus, increasing antigen size or display density does not necessarily result in a more effective vaccine and may instead compromise phage production or particle integrity.

Payload expression can also become a limiting factor even when the inserted genetic sequence can technically be accommodated within the phage genome. Recent studies of engineered bacteriophages have shown that the expression of heterologous genetic payloads can interfere with phage–host interactions and reduce phage fitness or progeny production [131,132]. In particular, inappropriate payload expression can disrupt the timing of phage gene expression, impose metabolic burdens on the bacterial host, or interfere with phage assembly, thereby compromising the recovery of viable recombinant phage particles. This illustrates an important trade-off between maximizing expression of an antigen-encoding cassette and preserving phage fitness during production. Controlled or tunable expression, appropriate genomic insertion sites, optimization of promoter strength, and computationally guided selection of payload configurations therefore represent potential strategies for reducing payload-associated production failures. However, these approaches require validation for individual phage–antigen combinations rather than being assumed to be universally applicable.

For phage DNA and hybrid vaccine platforms, an additional bottleneck occurs after administration. Delivery of phage-associated DNA to mammalian antigen-presenting cells does not necessarily result in efficient intracellular trafficking, nuclear localization, transcription, translation, or sustained antigen expression. The efficiency and duration of antigen expression can be influenced by several factors, including cellular uptake, intracellular trafficking and nuclear entry, promoter selection, DNA persistence, and the accessibility of the delivered genetic cassette to the host transcriptional machinery [132,133]. These constraints are important considerations for phage DNA and hybrid vaccine platforms, in which successful delivery of the genetic payload does not necessarily translate into efficient or sustained antigen production. Experimental studies have demonstrated that bacteriophage particles can be internalized by antigen-presenting cells, including dendritic cells and macrophages, and can subsequently induce antigen-specific humoral and cellular immune responses. However, the mechanisms governing phage uptake, intracellular trafficking, processing, and antigen presentation remain incompletely characterized and appear to depend on the phage architecture and cellular context. Studies with filamentous phages have shown that internalized particles can access both MHC class I and class II antigen-processing pathways, while other work has implicated endosomal/lysosomal trafficking and TLR9-dependent sensing of phage DNA in the activation of dendritic cells [134,135,136]. The relative contribution of phage DNA, capsid components, and the displayed or encoded antigen to the overall immune response therefore remains incompletely defined. These uncertainties are particularly relevant when extrapolating from animal models to humans, where differences in cellular uptake and pre-existing immunity may alter vaccine performance.

Several strategies could potentially address these constraints. These include the use of optimized eukaryotic promoters, regulatory elements that permit controlled rather than excessive expression, selection of genomic regions that minimize disruption of essential phage functions, and engineering of capsid proteins to accommodate antigen display without compromising particle assembly. For DNA-containing platforms, improving cellular uptake and intracellular trafficking may also be necessary to increase antigen expression. Nevertheless, the optimal configuration is likely to be antigen- and phage-specific, and there is currently insufficient evidence to define a universal payload threshold for phage-based vaccines against *S.* Typhi.

### 4.2. Manufacturing and GMP Challenges

Manufacturing is another major barrier to clinical translation. Most bacteriophages are amplified in bacterial hosts, commonly *Escherichia coli* or other susceptible bacteria, which provides a potentially scalable biological production system but introduces host-derived contaminants that must be removed before administration to humans. In particular, lipopolysaccharide (LPS) or endotoxin from Gram-negative production hosts represents a major safety concern because residual endotoxin can induce excessive inflammatory responses [105,106]. Consequently, production of a phage-based vaccine for human use requires downstream purification processes capable of substantially reducing endotoxin and other bacterial contaminants while maintaining adequate phage recovery, infectivity, and biological activity. This balance represents an important manufacturing challenge because aggressive purification can reduce product yield or compromise phage integrity [105,137].

Phage purification may involve combinations of clarification, filtration, precipitation, chromatography, ultrafiltration, and other downstream processes. However, each additional purification step can reduce product recovery and increase manufacturing complexity. This creates a practical tension between achieving pharmaceutical-grade purity and maintaining sufficiently high phage yields. In addition to endotoxin, manufacturing processes must control residual host–cell proteins, bacterial DNA, cellular debris, and other process-related impurities. The required purification strategy may also vary according to the phage, production host, formulation, and intended route of administration.

Scale-up introduces further challenges. A process that produces a reproducible phage preparation at laboratory scale does not necessarily maintain the same yield, purity, particle integrity, or potency when transferred to larger bioreactors [137,138,139,140]. Differences in host–cell physiology, phage replication kinetics, fermentation conditions, downstream recovery, and storage can contribute to batch-to-batch variability. Engineered phages introduce an additional consideration because genetic modifications must remain stable during repeated production cycles, and the final product must contain the intended phage genotype and antigenic construct.

Although phages possess long safety records and typically classify as BSL-1 agents, making Good Manufacturing Practice (GMP) production conceptually feasible [141], the existence of an established bacterial fermentation paradigm does not guarantee simple or inexpensive manufacturing. Clinical translation requires rigorous upstream and downstream processing, including validated purification, strict endotoxin removal, defined raw materials, and stringent release criteria, meaning actual production costs will ultimately depend on fermentation yield, downstream recovery, facility overhead, and batch consistency.

Potential solutions include the development of optimized low-endotoxin production hosts, improved chromatographic and membrane-based purification methods, validated removal of host-derived contaminants, standardized potency assays, and rigorous in-process and final-product quality control [107,108,109]. Process analytical technologies may also be required to ensure consistency between manufacturing batches. Importantly, these approaches remain largely platform-level solutions; their effectiveness specifically for a *S.* Typhi phage vaccine remains to be demonstrated.

### 4.3. Biosafety and Regulatory Considerations

Regulatory and biosafety requirements represent additional obstacles because phage-based vaccines have considerably less clinical and regulatory precedent than conventional protein, polysaccharide, or conjugate vaccines [133]. Although many bacteriophages are considered biologically distinct from mammalian viruses because they infect bacteria rather than human cells, the safety assessment of a vaccine product cannot be based solely on the natural biology of the parental phage. The characteristics of the final construct, including genetic modifications, antigen payload, production host, route of administration, and intended population, must be considered when determining the appropriate safety and regulatory requirements.

The regulatory complexity becomes greater when phages are genetically modified to display heterologous antigens or carry eukaryotic expression cassettes. Such products combine characteristics of biological vaccines, particulate delivery systems, and, in some cases, genetically modified biological entities. Consequently, developers may need to address requirements concerning identity, purity, potency, genetic stability, residual host-derived material, biodistribution, replication competence, and environmental safety.

For engineered phages, the genetic stability of the vaccine construct is particularly important [140]. A regulatory assessment would need to establish that the intended antigenic sequence remains stable during production and does not undergo unwanted rearrangement or loss. Where phages contain DNA intended for expression in mammalian cells, additional characterization of the genetic cassette and its biological activity would also be required. The presence of residual bacterial components, particularly endotoxin and host–cell DNA or proteins, would need to be controlled through validated manufacturing and purification procedures.

Environmental and biosafety considerations may also become relevant for genetically modified phages. Although bacteriophages generally have highly specific bacterial host ranges and do not replicate in mammalian cells, the deliberate administration or environmental release of engineered phages raises questions concerning their persistence, interaction with environmental bacterial populations, horizontal genetic exchange, and stability outside the intended host [102,142]. The importance of these considerations will depend on phage biology, genetic modifications, route of administration, and proposed use. They should therefore be evaluated on a case-by-case basis rather than assuming that all phage vaccine candidates present identical risks.

A further challenge is the absence of extensive human clinical experience with phage-based vaccines against *S.* Typhi. Regulatory authorities have accumulated experience with phage-based therapeutic products and other engineered biological vectors, but this experience does not automatically establish a standardized pathway for phage vaccines [143]. Developers will consequently need to generate comprehensive preclinical safety, pharmacological, immunogenicity, manufacturing, and quality data before clinical evaluation. Coordinated guidance from regulatory authorities and international public-health organizations could help establish consistent expectations for characterization, manufacturing, biosafety assessment, and clinical development.

### 4.4. Platform Failure Modes and Immunological Risks

Beyond manufacturing and regulatory considerations, phage vaccine platforms may fail at several stages between administration and protective immunity. One potential limitation is the development of anti-phage immune responses. Phage capsid proteins can themselves be recognized by the host immune system, and repeated administration may result in antibodies or other immune mechanisms capable of accelerating phage clearance [144]. If phage particles are neutralized or removed before sufficient antigen delivery occurs, the efficacy of subsequent doses could be reduced. This issue is particularly relevant to DNA and hybrid platforms in which successful delivery of the phage-associated genetic cargo is required for antigen expression.

The immune response to the carrier may also influence the response to the displayed or encoded antigen. Phage particles contain repetitive structural proteins and nucleic acids capable of stimulating innate and adaptive immune pathways [129,130]. Although this intrinsic immunostimulatory activity can be advantageous for vaccine development, it may also influence antigen processing and immune dominance. The relative contribution of carrier-specific and antigen-specific immunity may vary according to phage species, antigen configuration, particle dose, route of administration, and host immune status. Current evidence indicates that phage uptake, innate immune activation, antigen presentation, and immune polarization are not completely understood and may differ between phage platforms [144].

Structural instability represents another possible failure mode. In phage-display systems, insertion of large or structurally complex antigens into capsid proteins can interfere with protein folding, capsid assembly, particle stability, or accessibility of the displayed epitope [80,98]. Conversely, reducing antigen density to preserve phage fitness may decrease the amount of antigen presented to the immune system. An optimal balance between structural integrity and antigen density must therefore be determined experimentally for each construct.

Hybrid platforms introduce additional potential interactions between the two vaccine components. Surface-displayed antigen may generate a strong antibody response against the exposed antigen, while the DNA component is intended to generate intracellular antigen expression and potentially stronger cellular immunity. These responses could theoretically be complementary, but they could also interfere with one another. For example, antibodies recognizing the surface antigen or phage carrier could accelerate clearance of subsequent particles and thereby reduce delivery of the DNA cassette [135]. Conversely, immune responses generated against the encoded antigen could alter the persistence of particles displaying the same antigen. At present, there is insufficient experimental evidence to determine whether such interactions will be synergistic or antagonistic in a phage-based typhoid vaccine, making this an important area for future investigation.

Several strategies could potentially mitigate these failure modes. These include modifying capsid proteins to reduce unwanted immunodominance, selecting phages with appropriate host and structural properties, optimizing antigen density, using heterologous phage backbones for repeated administration, modifying administration schedules, and engineering particles to improve stability or cellular uptake. For DNA and hybrid platforms, optimization of the expression cassette and intracellular delivery pathway may further improve antigen availability. However, these interventions must be evaluated alongside their potential effects on phage production, immunogenicity, and safety.

### 4.5. Translational Outlook and Implications for Global Health

The potential relevance of phage-based vaccines extends beyond their immunological properties to questions of vaccine accessibility and manufacturing capacity in low- and middle-income countries (LMICs). Bacterial fermentation provides a potentially scalable foundation for phage production and could, where appropriate infrastructure and quality-control capacity are available, offer opportunities for regional manufacturing [12,14,15,16]. However, the actual economic advantage of such a system remains to be established because production costs will also depend on purification, endotoxin removal, formulation, quality testing, GMP compliance, and distribution requirements.

Similarly, the structural stability reported for some bacteriophages provides a rationale for investigating formulations with improved temperature tolerance. Such properties could eventually contribute to reducing cold-chain dependence, but reduced cold-chain requirements cannot currently be considered an established advantage of phage-based typhoid vaccines. Stability must be demonstrated for the specific phage construct and final formulation under realistic storage and transportation conditions before such an advantage can be translated into deployment policy.

The modularity of phage platforms may nevertheless provide an important developmental advantage. Antigens can potentially be modified, exchanged, or combined within established phage backbones, allowing iterative optimization of vaccine candidates as information about protective antigens and circulating *Salmonella* strains improves. This flexibility may be particularly valuable for typhoid fever, where current vaccines primarily target the Vi polysaccharide of *S.* Typhi and provide limited protection against *S.* Paratyphi and other causes of enteric fever. However, whether phage platforms can provide broader protection through multivalent antigen display or alternative antigen-selection strategies remains a research question rather than an established clinical benefit.

From a global-health perspective, therefore, the principal promise of phage-based vaccine platforms lies not in demonstrated superiority over existing typhoid vaccines, but in their potential combination of modular antigen engineering, particulate delivery, bacterial-host production, and adaptable platform design. These characteristics justify continued investigation, particularly where conventional vaccine technologies face limitations related to antigen breadth, duration of protection, manufacturing infrastructure, or delivery. Nevertheless, meaningful contributions to vaccine equity and antimicrobial-resistance mitigation will depend on demonstrating safety, protective efficacy, manufacturing feasibility, affordability, and deployability in settings where typhoid burden is highest. To contextualize emerging technologies against established standards, Table 4 provides a comparative synthesis of conventional and preclinical typhoid vaccine platforms, contrasting their developmental maturity, routes of administration, target populations, immunological mechanics, duration of protection, and global health accessibility profiles.

At present, major research gaps remain. No phage-based vaccine targeting *S.* Typhi or *S.* Paratyphi has progressed to human clinical evaluation, and most supporting evidence derives from preclinical studies involving other pathogens or from phage engineering and therapeutic research. Future studies should therefore prioritize *S.* Typhi-relevant antigen selection and validation, comparative evaluation of display and DNA-delivery architectures, standardized immunogenicity and protection endpoints, formulation and stability testing, GMP-compatible manufacturing, and systematic biosafety assessment. Collaborative research involving academic laboratories, manufacturers, regulatory agencies, and public-health institutions will be necessary to determine whether the theoretical advantages of phage platforms can ultimately be translated into a safe, effective, affordable, and globally deployable typhoid vaccine.

## 5. Conclusions and Future Directions

While bacteriophage-based platforms present a versatile and engineering-driven alternative to conventional typhoid vaccines, the field currently remains at a critical translational stage. To progress beyond platform-level proof-of-concept, future studies must establish not only immunogenicity but also protective efficacy using animal models that appropriately reflect the biology of *Salmonella enterica* serovar Typhi. Mouse models are particularly valuable for early-stage evaluation because they permit controlled assessment of vaccine dose, administration route, antigen formulation, immune responses, bacterial burden, and survival. However, conventional mice infected with *S.* Typhimurium provide an imperfect surrogate for human typhoid because *S.* Typhimurium and *S.* Typhi differ in host range, tissue tropism, and disease mechanisms [156,157]. Accordingly, such models should primarily be used to assess platform immunogenicity and protective mechanisms rather than interpreted as complete models of human typhoid.

For more pathogen-specific evaluation, humanized mouse models capable of supporting *S.* Typhi infection provide an important complementary approach. Human immune-system-engrafted mice have been shown to support *S.* Typhi replication and persistence and to generate human immune responses, offering a potentially more relevant system for evaluating candidate typhoid vaccines [158,159]. Other experimental models, including susceptible mouse systems and selected non-murine models, may provide additional information on mucosal immunity, systemic dissemination, and vaccine-mediated protection, although each has important limitations. The choice of model should therefore be matched to the specific development question. Early studies could use conventional mouse models to compare phage constructs, doses, and routes of administration, followed by more pathogen-relevant models to investigate protection against *S.* Typhi. For candidate oral vaccines, particular emphasis should be placed on intestinal and mucosal endpoints, including secretory IgA, mucosal T-cell responses, intestinal colonization or dissemination, and systemic bacterial clearance.

A rational preclinical program should consequently progress from immunogenicity to functional protection using standardized comparisons of phage display, phage DNA, and hybrid constructs. Studies should measure antigen-specific antibody titers and functionality, CD4+ and CD8+ T-cell responses, memory responses, mucosal immunity where relevant, bacterial burden in systemic and target tissues, and survival or other validated protection endpoints. Such studies would provide substantially stronger evidence for advancement than antibody titers alone and would help identify which phage architecture, antigen combination, and delivery route is most appropriate for typhoid vaccination.

## Figures and Tables

**Table 1:** Summary of clinical trials evaluating licensed typhoid vaccines by country, formulation, and immunogenicity outcomes.

Vaccine	Country	Trial Phase	Route	Mean Age (Years)	Doses	Outcome (Efficacy vs. Effectiveness)	Reported Side Effects	References
Ty21a	UK	II	Oral	39	3 ^1^	35.0% ^2^	Headache, malaise, abdominal pain	[41]
Ty21a	Chile	II	Oral	12.5	3	62.0% ^3^	NR	[35]
Ty21a	Indonesia	II	Oral	23.5	3	42.2% ^3^	Nausea, headache, diarrhea	[42]
TCV	Nepal	III	Intramuscular	8.4	1	79.0% ^3^	Headache, fatigue	[59]
TCV	Malawi	III	Intramuscular	6.4	1	78.3% ^3^	No serious adverse effects	[34]
ViPS	UK	II	Intramuscular	39	1	54.6% ^2^	Low-grade fever	[78]
Vi-rEPA	Vietnam	I and II	Intramuscular	3.5	2	91.5% ^3^	Low-grade fever, local swelling	[79]

^1^ Standardized as number of doses; regimens typically administered on days 1, 3, and 5. ^2^ Measured as vaccine efficacy via human challenge model. ^3^ Measured as vaccine effectiveness in field trials.

**Table 2:** Comparative characteristics of major phage-derived vaccine platforms relevant to typhoid vaccine development.

Platform	Display Capacity/Antigen Architecture	Payload Capacity	Immunogenicity	Production	Stability	Suitability for Oral/Mucosal Delivery
T4	Large icosahedral capsid; Hoc and Soc provide multiple non-essential display sites; suitable for peptides and relatively large proteins	Large dsDNA genome (~171 kb) provides substantially greater genetic space than small ssRNA phage genomes, although functional payload capacity remains construct-dependent	Strong particulate immunogenicity; can induce humoral and cellular responses; mucosal responses demonstrated in non-typhoid models	Propagated efficiently in bacterial hosts; established fermentation and purification approaches	Relatively robust capsid; structural stability can be maintained with appropriately engineered display constructs	Promising; mucosal and needle-free T4 vaccine studies support feasibility, but oral gastrointestinal delivery for typhoid requires direct validation
M13	Filamentous phage; pVIII (~2700 copies) supports high-density peptide display; pIII is suitable for larger fragments	~6.4 kb ssDNA genome; useful for phage-display constructs but less suitable for large genetic payloads	Strong repetitive antigen presentation; extensive evidence for antibody and cellular responses	Well-established *E. coli* propagation and phage-display workflows; comparatively simple experimental production	Filamentous architecture is physically robust, but large fusion proteins may affect assembly and secretion	Potentially suitable; needle-free and mucosal applications have been explored, but gastrointestinal stability and uptake require specific validation
Qβ	~28 nm VLP with ~180 coat proteins; suitable for peptide display and chemical conjugation of larger antigens	~4.2 kb ssRNA genome in parental phage; final VLP vaccine generally functions as a non-replicating particulate scaffold	Strong humoral immunogenicity; repetitive antigen arrangement supports B-cell activation; extensive VLP vaccine experience	Self-assembly in *E. coli*; established expression and purification methods	Compact, highly ordered capsid; VLP architecture can provide substantial protection to associated antigenic material	Potentially suitable as a mucosal particulate vaccine, but gastrointestinal stability, uptake, and appropriate adjuvantation must be established for oral typhoid vaccination
AP205	~27–30 nm VLP with ~180 coat proteins; supports genetic fusion and modular antigen attachment	~4.3 kb ssRNA genome in parental phage; final VLP is generally used as a non-replicating scaffold	Immunogenic particulate platform; can support repetitive antigen presentation and innate immune stimulation	Self-assembling VLP production with established bacterial expression and purification approaches	Compact capsid architecture; stability depends on antigen design and formulation	Promising but unproven for oral typhoid vaccination; particularly relevant because *S.* Typhi antigens have already been displayed on AP205 particles
Relative interpretation	T4 offers the greatest structural flexibility for large/multicomponent displays; M13 favors high-density peptide presentation; Qβ and AP205 favor compact VLP-based particulate presentation	Genome size does not directly equal usable vaccine payload capacity; functional limits depend on phage biology, insertion site, expression burden, and particle assembly	All can be immunogenic, but magnitude and quality of responses are construct-, antigen-, dose-, and route-dependent	All have established laboratory production systems, but GMP-scale manufacture and purification remain platform-specific challenges	No platform should be assumed to be universally superior; formulation and construct design strongly influence stability	Oral suitability remains an experimental question and should be assessed using gastrointestinal stability, mucosal uptake, SIgA, cellular immunity, and protection endpoints

**Table 3:** Comparative characteristics, potential advantages, and challenges of phage-based and other particulate vaccine platforms relevant to typhoid vaccine development.

Vaccine Platform	Core Characteristics	Potential Advantages	Key Challenges	Relevance to Typhoid Vaccine Development
Phage-based vaccines	Bacteriophage particles engineered for antigen display, nucleic-acid delivery, or combined/hybrid approaches	Genetic programmability; multivalent antigen presentation; modular surface engineering; potential combination of humoral and cellular antigen-delivery strategies; bacterial propagation may support scalable production	Endotoxin contamination; anti-phage immunity and particle clearance; payload and capsid-assembly constraints; genetic stability; limited clinical and regulatory precedent for typhoid vaccines	Promising but currently experimental; no phage-based vaccine specifically targeting typhoid fever has yet demonstrated protective efficacy in preclinical or clinical studies
Virus-like particles (VLPs)	Non-infectious, genome-free particles formed by self-assembly of viral structural proteins or related scaffolds	Highly ordered and repetitive antigen presentation; strong immunogenicity; defined particle architecture; established clinical and commercial precedent for several vaccines	Production and scale-up complexity; particle assembly; antigen incorporation; upstream/downstream processing; product-quality control	Relevant precedent for multivalent particulate presentation; AP205-based *S.* Typhi antigen display illustrates the potential of VLPs for organizing multiple antigens
Outer membrane vesicles (OMVs)	Naturally derived vesicles released from Gram-negative bacteria and containing membrane-associated proteins, lipids, and immunostimulatory components	Intrinsic immunostimulatory/self-adjuvanting properties; presentation of multiple bacterial antigens; potential for genetic engineering; established clinical experience with some OMV-based vaccines	LPS-associated reactogenicity; antigen heterogeneity; variable production yield; purification and standardization; heterologous antigen loading; long-term stability	Particularly relevant to bacterial vaccine development and multicomponent antigen delivery, but safety and composition require careful control
Synthetic/protein-based nanoparticles	Engineered nanoscale particles with tunable size, composition, antigen density, orientation, and surface properties	Precise control of antigen organization; multivalent presentation; potential optimization of lymphatic trafficking, APC uptake, and B-cell receptor engagement	Formulation and manufacturing complexity; control of particle size and antigen density; batch consistency; stability; scale-up and storage	Provides a highly tunable alternative for antigen presentation and immunofocusing, but requires substantial optimization for pathogen-specific protection

**Table 4:** Comparison of conventional and emerging typhoid vaccine platforms by immunological characteristics, protection duration, and accessibility.

Vaccine Type	Development Stage	Level of Evidence	Delivery Method	Age Indication	Immune Response Type	Duration of Protection	Cost and Accessibility	References
ViPS	Licensed	Clinical/Human Data	Single IM injection	≥2 years	T-cell independent; weak booster	2–3 years	Relatively low cost; widely available	[47,145,146]
TCV	Licensed	Clinical/Human Data	Single IM injection	≥6 months	T-cell dependent; strong booster	4–6 years	Higher upfront cost; limited rollout	[147,148,149]
Ty21a	Licensed	Clinical/Phase III and field-trial data	3–4 oral doses (capsules)	≥5 years	Induces mucosal and systemic immunity	~5 years (variable)	Moderate cost; cold-chain dependent	[150,151,152]
Phage-based ‡	Preclinical	Preclinical/proof-of-concept; no human typhoid vaccine data	Potentially oral or parenteral; route-dependent and not yet established for typhoid	N/A (Preclinical only)	Innate, humoral and cellular responses demonstrated in platform studies ^1^	Not yet established	Projected scalability and potential thermostability ^2^	[20,103,104,153,154,155]

Abbreviations: ViPS—Vi Polysaccharides; TCV—Typhoid Conjugate Vaccine; N/A—Not Applicable. ‡ Denotes platforms currently in the discovery or preclinical stage of development. ^1^ Based on animal immunogenicity and proof-of-concept data; clinical performance in humans is not yet established. ^2^ Projected cost based on the scalability of bacterial culture systems and independence from cold-chain infrastructure.

## Data Availability

All the data required to support the conclusions are presented in this paper.
